# 

**Published:** 1893-03-18

**Authors:** 


**OUR NEW OFFICES.**

428, Strand, W.C., will henceforth be the Office of this Journal.

### NOTICE TO CORRESPONDENTS.

All MS., Letters, Books for Review, and other matters intended for the Editor should be addressed The Editor, The Lodge, Porchester Square, London, W.

The Editor cannot undertake to return rejected M.S., even when accompanied by stamped directed envelope.

**Notice to Advertisers and Subscribers.**

All Advertisements, Orders for Copies of The Hospital, and Business Communications should be addressed to The Publisher, *not to the Editor*, The Hospital, 428, Strand, W.C.

The Cost of Subscription, post free, is as follows:—Per week, 2½d.; per quarter, 2s. 6d.; per half-year, 5s.; per annum, 8s. 6d. Foreign:—Per annum, 10s. 8d.; payable, in all cases, in advance.

**"Burdett's Hospital Annual," 1893.**

Fourth year of publication. 700 pp. Boards, gilt lettering. A most complete guide to British, Colonial, Indian, and American Hospitals, Dispensaries, Nursing and Convalescent Institutions, and Asylums. Price 5s. Published by The Scientific Press (Limited), 428, Strand, W.C.

**NOTICE.—The Index for Vol. XII. (ending September 1892) is on sale at the Office of this Journal, price 2½d., post free.**

### SCRAPS AND GLEANINGS.

A TEMPORARY infectious diseases hospital is to be shortly provided for Denbigh.

THE Queen has contributed £50 towards the Queensland Floods Relief Fund, which fund amounts now to over £6,000.

SMALL-POX has broken out in Japan. There were 340 cases reported from Kobé in February, 149 of which proved fatal.

THE Marquis of Salisbury has presented a cheque of £100 towards the new building fund of the Radcliffe Infirmary, Oxford.

MR. BRADSHAW has sent a cheque of £50 towards the building fund of the proposed convalescent home for children at Nottingham.

THE Princess of Wales has forwarded £50 to the Lord Mayor, for the relief fund of the sufferers by the recent disastrous earthquake at Zante.

BY the death of Sir Andrew Walker, Liverpool has lost a great benefactor. The deceased is estimated to have been possessed of 3,000,000 sterling.

INFLUENZA being very prevalent in the Chatham and Rochester district in early February, the medical officer of health ordered a temporary closing of the day and Sunday schools.

WE regret that a mistake occurred in our issue of the 25th ult. in reference to the Aberdeen Infirmary. It should have been stated that the Convalescent Home in connection with the Infirmary is in so bad a financial condition that it is proposed to close it.

THE danger of placing flower-pots outside windows was tragically proved in Paris the other day, when one falling on the head of a gentleman caused fracture of the skull. He was conveyed to the St. Louis Hospital, where he succumbed to the injuries in a few hours.

For Student of Medicine and Medical Appointments see pages X. and XI. overleaf.

### FIRST IMPRESSIONS OF BOOKS RECEIVED.

[It is requested that all books intended to be noticed in this column may be sent to the Editor, at the Office, 428, Strand, W.C., not later than Tuesday evening in each week.]

We note among the publications of the week a third edition of P. G. Lewis's "Theory and Practice of Nursing" (Scientific Press), J. S. Cassidy's "Elements of Chemistry and Dental Materia Medica," and Dr. A. A. Stevens' "Manual of the Practice of Medicine."

Among the books to be presently published by Longmans and Co. are "Essays on Rural Hygiene," by Dr. Vivian Poore, who is sure to treat the subject with freshness and ability; two books on "Hernia," by Mr. W. H. Bennett and Mr. W. Longton; and a translation by Dr. Dawson of Professor Schersk's "Elements of Bacteriology." This is the most recent German work on the subject, and deals with it on its practical side.

"Sir Morell Mackenzie, Physician and Operator," by the Rev. H. R. Haweis, M.A. (Allen and Co.), and a volume of "Essays by Sir Morell Mackenzie," edited by his brother, A. W. Mackenzie (Sampson Low), will appear shortly.

Among other announcements of books we note the following: "Physical Education," by F. Treves, a reprint from "A Treatise on Hygiene"; a second edition of Gowers' Lectures on "Syphilis and the Nervous System: An Introduction to the Study of Diseases of the Skin," by Dr. Pye-Smith; and the third edition of Dr. Taylor's "Manual of the Practice of Medicine." (Churchill). "How to Become a Hospital Nurse," by Alice Dannatt, and "Dispensing for Nurses," by C. J. S. Thompson. (The Record Press.) Migula's "Practical Bacteriology," translated by Dr. Campbell, of Guy's Hospital; "Text Book of Embryology," by Korschelt and Heider, of Berlin, translated by Drs. Mark and Woodward, of Harvard University; "The Cell, its Anatomy and Physiology," by Oscar Hertwig, also translated by Dr. Campbell, of Guy's; and three works on Botany, by Professor Vines, of Oxford; Dr. Warming, of Stockholm, translated by Mr. Potter; and Professor Strasburger, of Bonn, translated by Professor Hillhouse; "A Popular History of Medicine," Dr. Berdoe. (Swan Sonnenschein.)

Dr. Arthur Newsholme's translation of Albert Palmberg's "Treatise on Public Health" is published. In addition to chapters on the Sanitation of England, and London in particular, it gives similar information as to France, Sweden, Germany, Austria, and Finland. The book is published by Swan Sonnenschein and Co.

Bidgood's "Course of Practical Elementary Biology" (Longmans), one of the Elementary Science Manuals, is a guide for beginners which covers the ground of most elementary biological courses.

"A Manual of Health and Temperance." (Longmans.) To chapters written by Mr. Bradribb, Secretary to the Education Department of Victoria, the Rev. W. Ruthven Pym has added selected passages from Gough's orations. It is addressed to children.

### THE STUDENT OF MEDICINE.

[All communications for this Supplement should be addressed to The Editor of The Hospital, at 428, Strand, with the words, "Students' Column," written in the left hand top corner of the envelope.]

### APPOINTMENTS.

Gervase E. Newby, M.R.C.S.Eng., L.R.C.P.Lond., appointed House Surgeon to the Salford Royal Hospital. Stanley M. Brown, M.R.C.S.Eng., L.R.C.P.Lond., appointed Junior House Surgeon to the Salford Royal Hospital. J. W. H. Allott, M.B., appointed Honorary Surgeon to the Beckett Hospital, Barnsley. B. Anningson, B.A.Cantab, M.D., M.R.C.S., reappointed Medical Officer of Health for Chesterton. V. E. Barrow, L.R.C.P., L.R.C.S., appointed Medical Officer to the Uffculme Foresters. W. J. Townsend Barker, L.R.C.P.Lond., M.R.C.S.Eng., appointed Medical Officer and Public Vaccinator for the No. 2 District Parish of Streatham of the Wandsworth and Clapham Union. C. J. Bernard, L.A.H., L.M.Dub., appointed Medical Officer to the Nottingham Convalescent Home. R. Bevan, L.R.C.P., D.P.H.Lond., reappointed Medical Officer of Health for the Ashford Urban Sanitary Authority. R. J. Blackham, L.R.C.P., L.R.C.S.Edin., L.F.P.S.Glasg., L.M.Rotunda Hospitals, F.R.M.C.S., appointed Physician to the Sidcup Hospital, London, S.E. J. R. Burnett, M.B., C.M.Edin., M.R.C.S.Eng., appointed House Physician to the Hospital for Consumption and Diseases of the Chest, Brompton. W. J. Butler, L.R.C.P.Lond., M.R.C.S. Eng., reappointed Medical Officer to the Nottingham Convalescent Home. M. P. M. Collier, M.S.Lond., F.R.C.S., appointed Surgeon to the National Hospital for Diseases of the Heart, &c., Soho Square, W. C. S. De Segundo, L.R.C.P.Lond., M.R.C.S.Eng., appointed House Surgeon to the London Temperance Hospital, Hampstead Road, N.W. N. C. Dobson, F.R.C.S., appointed Consulting Surgeon to the Bristol General Hospital. J. O'Conor Donelan, L.R.C.S.I., L.L.Mid., K.Q.C.P.I., appointed Assistant Medical Officer to the Richmond District Lunatic Asylum, Dublin. J. G. Duncanson, M.B., C.M.Glasg., appointed Resident Assistant to the Victoria Infirmary, Glasgow. W. T. Gardner, M.B.Lond., L.R.C.P., M.R.C.S., appointed Medical Officer of Health for the Aston Urban Sanitary District of the Brentford Union. A. E. Giles, M.D., B.Sc.Lond., M.B., B.Ch.Vict., M.R.C.P., M.R.C.S., appointed Physician-Accoucher to the St. Pancras and Northern Dispensary, Euston Road, N.W. W. A. Haslam, L.R.C.P. Lond., M.R.C.S.Eng., appointed Junior House Surgeon to the London Temperance Hospital, Hampstead Road, N.W. A. E. Hine, M.R.C.S.Eng., L.R.C.P.Lond., appointed Resident Obstetric Officer to Charing Cross Hospital, London. D. L. Hubbard, M.B.Durh., B.S., M.R.C.S., reappointed Medical Officer of Health for Lynton. W. L. Hunter, M.D., reappointed Medical Officer of Health for Pudsey. J. F. Jordan, M.B., B.Ch.Irel., F.R.C.S., L.R.C.P.Lond., appointed Surgeon to the Birmingham and Midland Counties Hospital for Women. B. Lumley, M.R.C.S.Eng., L.S.A., reappointed Medical Officer of Health to the Northallerton Local Board. S. G. Moore. M.B.Vict., Ch.B., appointed Assistant Port Sanitary Medical Officer for Liverpool. T. Morgan, L.R.C.P., I.R.C.S.I., reappointed Medical Officer for the Berriew District of the Forden Union. C. A. Morton, L.R.C.P.Lond., F.R.C.S., appointed Assistant Surgeon to the Bristol General Hospital. C. Munden, M.R.C.S., reappointed Medical Officer for the Third Ilminster Sanitary District of the Chard Union. Dr. J. J. Murphy, appointed Resident Medical Officer to the Cork Union Workhouse. W. Nettle, M.R.C.S.Eng., L.S.A., reappointed Medical Officer of Health to the Liskeard Rural Sanitary Authority. H. Cooper Pattin, M.A., D.P.H., M.B.Cantab., appointed Medical Officer of Health for the City of Norwich, and not York, as stated last week. N. Raw, M.D.Durh., M.B., B.S., L.S.Sc., appointed Medical Superintendent of the Dundee Royal Infirmary. J. Robertson, M.D.Edin., B.Sc., appointed Borough Analyst for the St. Helens Town Council. T. Savage, M.D., M.R.C.P.Lond., F.R.C.S.Eng., appointed Professor of Gynæcology in Mason College, Birmingham. J. W. Stoker, L.R.C.P., L.R.C.S.Edin., L.F.P. and S. Glasg., appointed Certifying Surgeon under the Factories Act for the District of Rainham, Essex, also Medical Examiner for Post Office Insurance for the same district. J. H. Vinrace,

### MEDICAL APPOINTMENTS.

THE GENERAL HOSPITAL, BIRMINGHAM.

PATHOLOGIST.

HOWARD J. COLLINS,
House Governor.

March 3rd, 1893. (1,123)

THE GENERAL HOSPITAL, BIRMINGHAM.

ROYAL HOSPITAL for DISEASES of the CHEST,

City Road, E.C.

There is a VACANCY for a HOUSE PHYSICIAN. Appointment for six months from April 1st. Salary at the rate of £40 per annum, and Board and Lodging.

Applications, with copies of testimonials, to be sent to the Secretary not later than the 23rd inst.

By order,
JOHN HARROLD,

(1,131) Secretary.

THE GENERAL HOSPITAL, BIRMINGHAM.

SOUTH DEVON AND EAST CORNWALL
HOSPITAL, Plymouth.

SEAMEN'S HOSPITAL SOCIETY,

"Dreadnought."

WANTED, SENIOR and JUNIOR HOUSE SURGEONS for Branch Hospital, Royal Victoria and Albert Docks, E.

Salaries, £75 and £50 per annum, with Board and Residence.

Candidates must be doubly qualified and registered.

Applications, and copies of not more than three testimonials, to be sent in on or before TUESDAY, 21st March, to the undersigned, from whom further particulars can be obtained.

By order,

P. MICHELLI, Secretary.

Seamen's Hospital Society,

Greenwich, S.E.,

March 6th, 1893.

(1,010)

### THE STUDENT OF MEDICINE—(*continued*).

M.B.Lond., M.R.C.P., appointed Physician to the National Hospital for Diseases of the Heart, &c., Soho Square, W. M. Young, M.B., C.M.Edin., appointed Medical Officer of Health for the Brighouse Urban Sanitary District and Rastrick Sanitary District of the Halifax Union.

### VACANCIES.

The following vacancies are announced this week, the amount of salary in each case being given after the name of the institution:

#### Resident Medical Officers.

Cancer Hospital (Free), Fulham Road, S.W. House Surgeon. Appointment for six months. Salary at the rate of £50 per annum, with board and residence. Applications to the Secretary by March 18th

Dental Hospital of London, Leicester Square, W.—Assistant Anæsthetist. Applications to J. Francis Pink, Secretary, by March 20th.

General Hospital, Birmingham.—Assistant House Surgeon. Board, residence, and washing provided. Applications to Howard J. Collins, House Governor, by April 1st.

Hartlepools Hospital. House Surgeon. Salary £60 per annum, with board, lodging, and washing. Applications to Robert Edger, Honorary Secretary, by March 20th.

Liverpool Infirmary for Children, Myrtle Street, Liverpool. House Surgeon. Salary £85 per annum, with board and lodging. Applications to C. W. Carver, Honorary Secretary, by March 18th.

London County Asylum, Claybury, Woodford, Essex. Second Assistant Medical Officer, unmarried, not more than 35 years of age, doubly qualified. Salary £180 per annum, rising by £10 a year to £230, with board, lodging, and washing. Applications, on forms to be obtained of the Clerk, to R. W. Partridge, Clerk to the Asylums Committee, 21, Whitehall Place, S.W., by March 20th.

Manchester Royal Infirmary. Assistant Medical Officer at the Monsall Fever Hospital, unmarried. Appointment for twelve months. Salary £100 per annum, with board and residence. Applications to the Chairman of the Board, Royal Infirmary, Manchester, by March 21st.

National Hospital for Consumpton, Ventnor. Dispenser, unmarried. Salary £60 per annum, with board and residence. Applications to the Secretary, 34, Craven Street, Charing Cross.

St. Peter's Hospital for Stone and Urinary Diseases, Henrietta Street, Covent Garden, W.C. House Surgeon. Appointment for six months. Honorarium 25 guineas, board, lodging, and washing. Applications to Walter E. Scott, Secretary, by March 21st.

Seamen's Hospital Society. Senior House Surgeon for Branch Hospital, Royal Victoria and Albert Docks, E.; doubly qualified. Salary £75 per annum, with board and residence. Applications to P. Mitchelli, Secretary Seamen's Hospital Society, Greenwich, by March 21st.

Seamen's Hospital Society. Junior House Surgeon for Branch Hospital, Royal Victoria and Albert Docks, E.; doubly qualified. Salary £50 per annum, with board and residence. Applications to P. Michelli, Secretary Seamen's Hospital Society, Greenwich, by March 21st.

Sheffield Children's Hospital (East End Branch), Sheffield. House Surgeon; doubly qualified. Salary £70 per annum, with board, lodging, and washing. Applications to the Honorary Secretary by March 30th.

South Devon and East Cornwall Hospital, Plymouth. Assistant Secretary; not under 35 years of age. Salary £150 per annum. Applications to J. Walter Wilson, Honorary Secretary, by March 25th.

West London Hospital, Hammersmith Road, W. House Surgeon; appointment for six months. Board and lodging provided. Applications to R. J. Gilbert, Secretary Superintendent, by March 22nd. Election on March 27th.

Worcester General Infirmary. House Surgeon; doubly qualified. Salary £100 per annum, with board and residence. Appointment for three years. Applications to William Stallard, Secretary, Worcester Chambers, Pierpoint Street, Worcester, by March 25th.

#### Non-Resident Medical Officers.

Bedford General Dispensary.—Dispenser, under 45 years of age. Salary £70 per annum. Applications to the Secretary by March 22nd.

General Hospital, Birmingham. Pathologist. Salary £120 per annum. Applications to Howard J. Collins, House Governor, by April 1st.

Liverpool Royal Infirmary. Honorary Laryngologist and Honorary Dermatologist. Applications to the Chairman of the General Committee by March 20th.

London Hospital, Whitechapel Road, E. Surgical Registrar. Salary £100 per annum. Applications to the House Governor by March 23rd.

Mason College, Birmingham, Co-Professor of Surgery. Applications to G. H. Morley by April 15th.

St. Marylebone General Dispensary, 77, Welbeck Street, Cavendish Square, W. Honorary Surgeon. Applications to the Secretary by March 22nd.

St. Marylebone General Dispensary, 77, Welbeck Street, Cavendish Square, W. Honorary Physician. Applications to the Secretary by March 22nd.

